# Supplementary material for: Specificity of the Hox member Deformed is determined by transcription factor levels and binding site affinities
Source: Nat Commun. 2022 Aug 26;13:5037. doi: 10.1038/s41467-022-32408-8 (PMC9418327; doi:10.1038/s41467-022-32408-8)
Supplement: Supplementary file 1 — Supplementary Information [file 41467_2022_32408_MOESM1_ESM.pdf]

Supplementary Information

Specificity of the Hox member Deformed is determined by transcription factor levels and binding site affinities

Pedro B. Pinto, Katrin Domsch, Xuefan Gao, Michaela Wölk, Julie Carnesecchi, Ingrid Lohmann

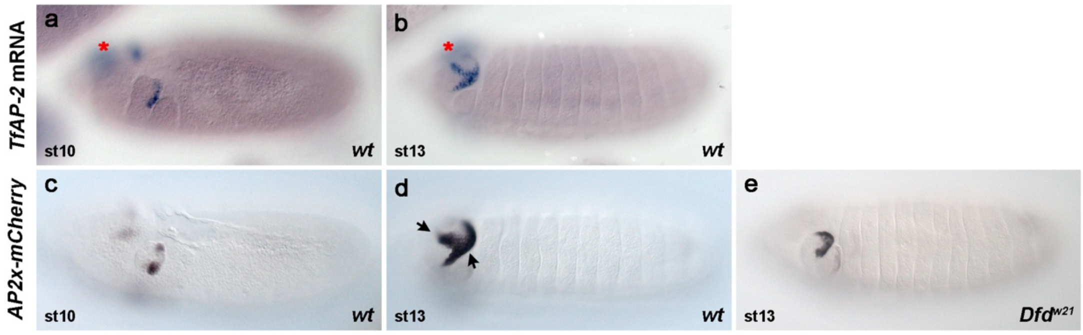

Supplementary Figure 1. The *AP2x* enhancer under the control of Deformed directs the expression of *AP-2* in the maxillary segment.

(a, b) *In situ* hybridisation of *AP-2* mRNA. *AP-2* is first expressed in the maxillary segment in stage 10 (a), full activation is achieved by stage 13 (b). **(c, d)** Activity of the *AP2x* maxillary enhancer. Embryos carrying the *AP2x-mCherry* reporter were stained for mCherry to determine the activity of *AP2x*. The enhancer directs expression of mCherry in a pattern similar to the endogenous *AP-2* gene. *AP2x* is first activated in stage 10 (c), full activation is achieved by stage 13 (d). **(e)** Expression of *AP2x* in Dfd^{w21} homozygous embryos. As the endogenous *AP-2*, the activity of *AP2x* in the medial stripe and ventral posterior cells is dependent of Dfd, while activation of *AP-2* in the dorsal posterior cells does not require Dfd. The arrows in (d) indicate the cells that are dependent on Dfd to activate *AP2x* in the maxillary segment. The red asterisks in (a, b) highlight *AP-2* expression in the brain.

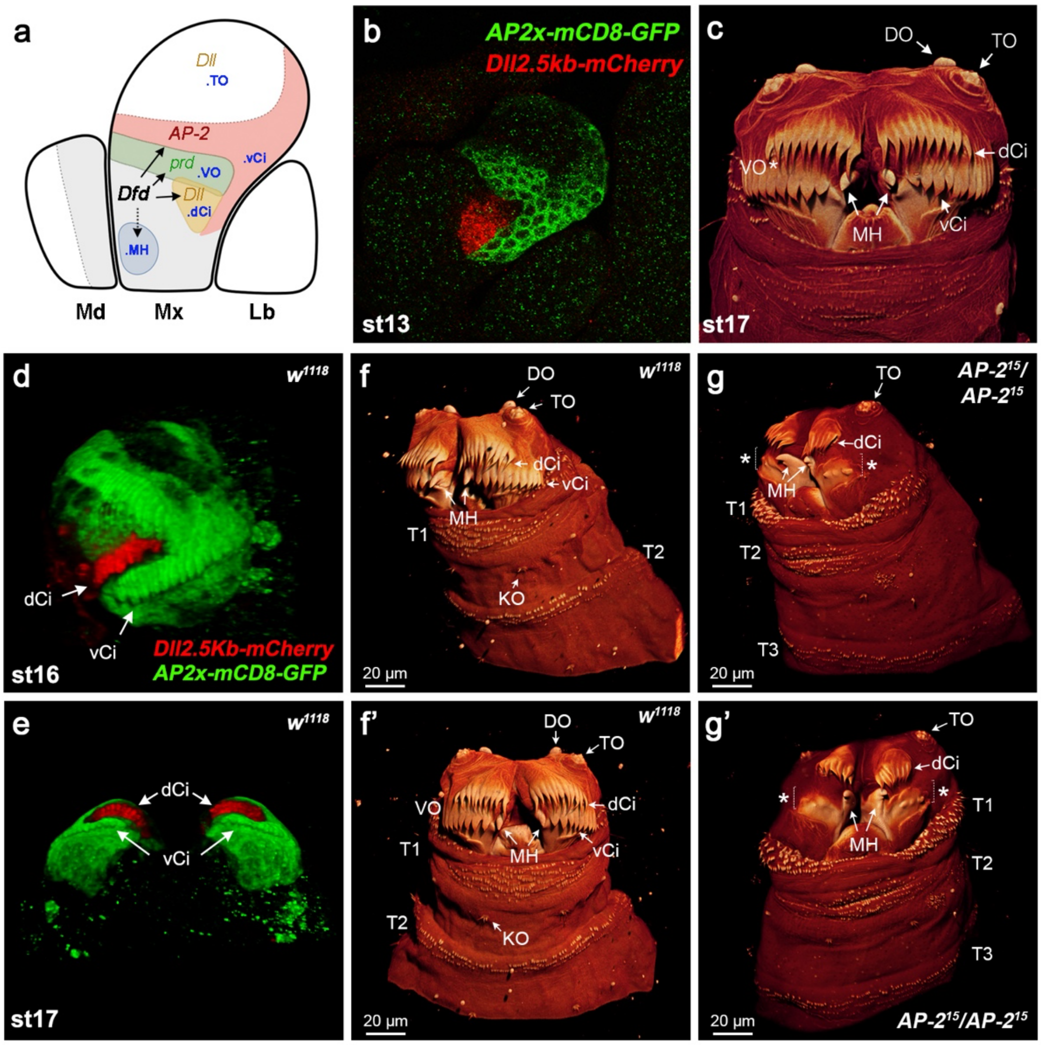

Supplementary Figure 2. The primordia of the dorsal and ventral cirri arise from different regions of the maxillary segment.

(a) Schematic representation of the gnathal segments and the maxillary segment cell-fate map. The Hox TF Dfd is expressed in the mandibular (Md) and maxillary (Mx) segments (grey area outlined by a dashed line). In the maxillary segment, Dfd activates, in specific domains, morphogenetic programs responsible for the development of specific maxillary structures: the Ventral Organ (VO; green), the dorsal cirri (dCi; yellow), the ventral cirri (vCi; pink) and the Mouth Hooks (MH; blue). The primordia of these structures have been characterised and targets of Dfd involved in the development of these structures have been identified: *paired* (*prd*) in the VO primordia, *Distal-less* (*Dll*) in the dCi primordia and *AP-2/tfAP-2* in the vCi primordia. **(b)** Expression pattern of the *AP2x-mCD8-GFP* reporter (green) and *Dll2.5Kb-mCherry* (red) in the maxillary segment of stage 13 embryos, highlighting the close vicinity of the primordia of dCi and vCi. **(c)** Anterior view of a 3D image of a wild-type *Drosophila* larva head. **(d, e)** Expression pattern of the *AP2x-mCD8-GFP* reporter (green) and

Dll2.5Kb-mCherry (red) in the maxillary segment of stage 16 (d) and 17 (e) embryos. (f-g') 3D images of the head structures of *Drosophila melanogaster* w^{1118} (f, f') and $AP\text{-}2^{15}$ homozygous (g, g') 1st instar larval cuticles: anterior view (f', g') and lateral view (f, g). The asterisks in (g, g') indicate the regions where vCi failed to develop in $AP\text{-}2^{15}$ homozygous mutants.

Mx: Maxillary segment; Lb: Labial segment; T1: 1st thoracic segment; T2: 2nd thoracic segment; DO: Dorsal Organ; TO: Terminal Organ; VO: Ventral Organ; dCi: dorsal cirri; vCi: ventral cirri; MH: Mouth Hooks; KO: Keilin's Organs.

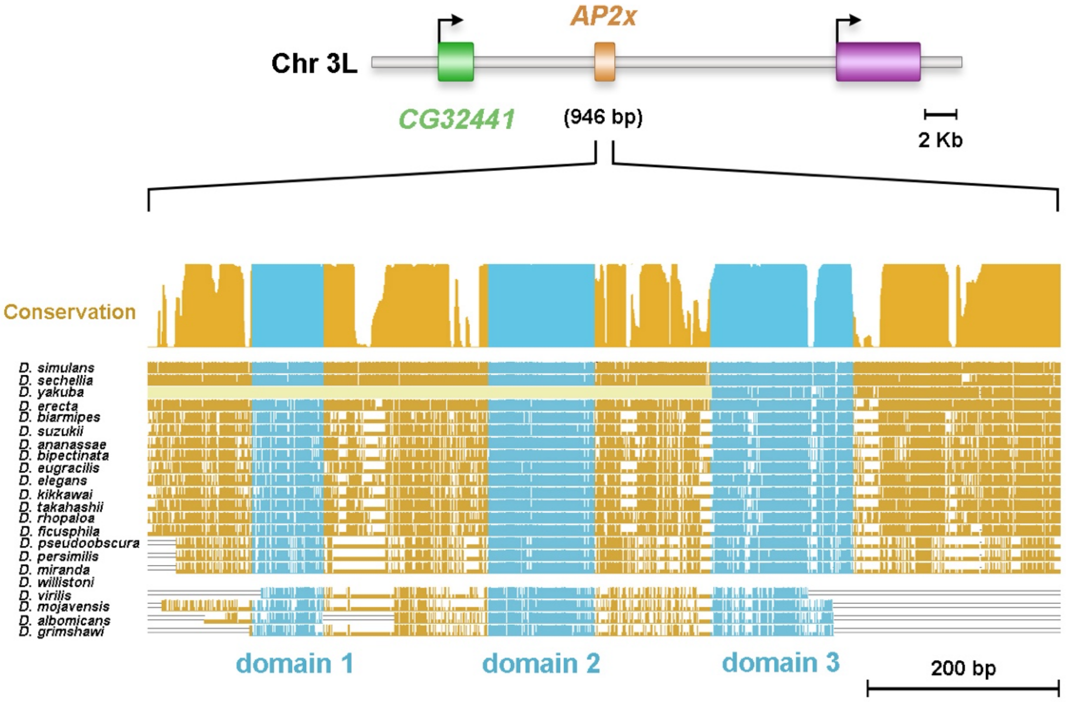

Supplementary Figure 3. The *AP2x* enhancer contains highly conserved domains.

Schematic representation of the *AP-2* locus and the conservation of *AP2x* enhancer element across different *Drosophila* species. The three regions in blue indicate highly conserved sequences.

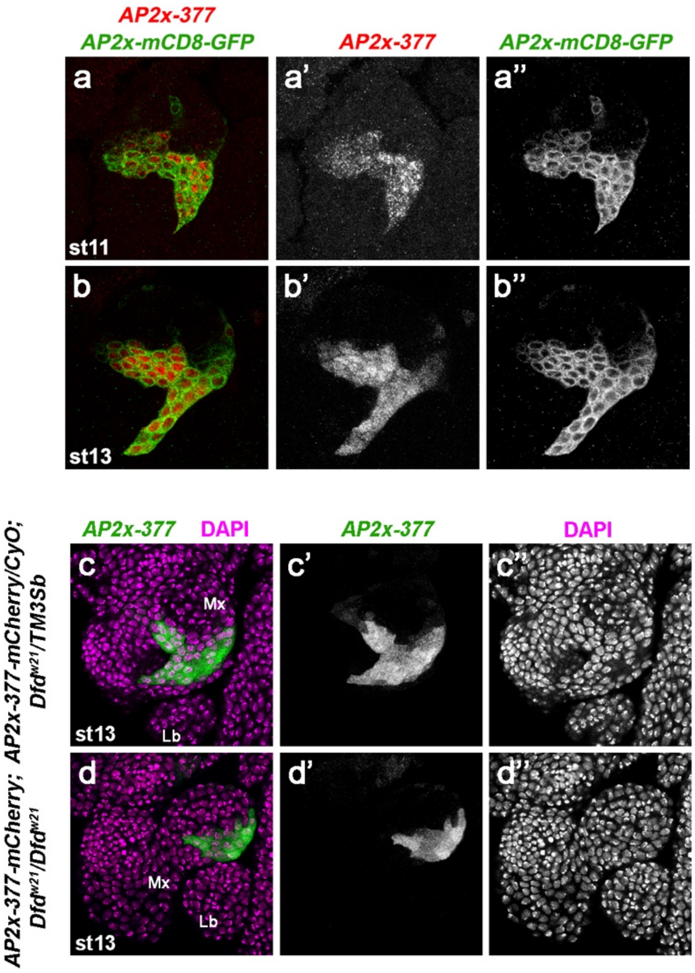

Supplementary Figure 4. **The *AP2x-377* enhancer contains the minimal information for maxillary activity.**

(a-b’’) Stage 13 embryos carrying both *AP2x-mCD8-GFP* and *AP2x-377-mCherry* reporters constructs stained for GFP (green in a and b, grey in a’’ and b’’) and mCherry (red in a, b and grey in a’, b’) to determine the spatial activation of *AP2x* and *AP2x-377* enhancers, respectively. **(c-d)** Activation of *AP2x-377-mCherry* in stage 13 *Dfd*^{w21} mutant embryos. *Dfd*^{w21} heterozygous (c-c’’) and homozygous (d-d’’) embryos were stained for mCherry to determine the activity of *AP2x-377* (green in c, d and grey in c’, d’); the maxillary segment was highlighted by co-staining embryos with DAPI (magenta in c, d and grey in c’’, d’’).

Mx: Maxillary segment; Lb: Labial segment.

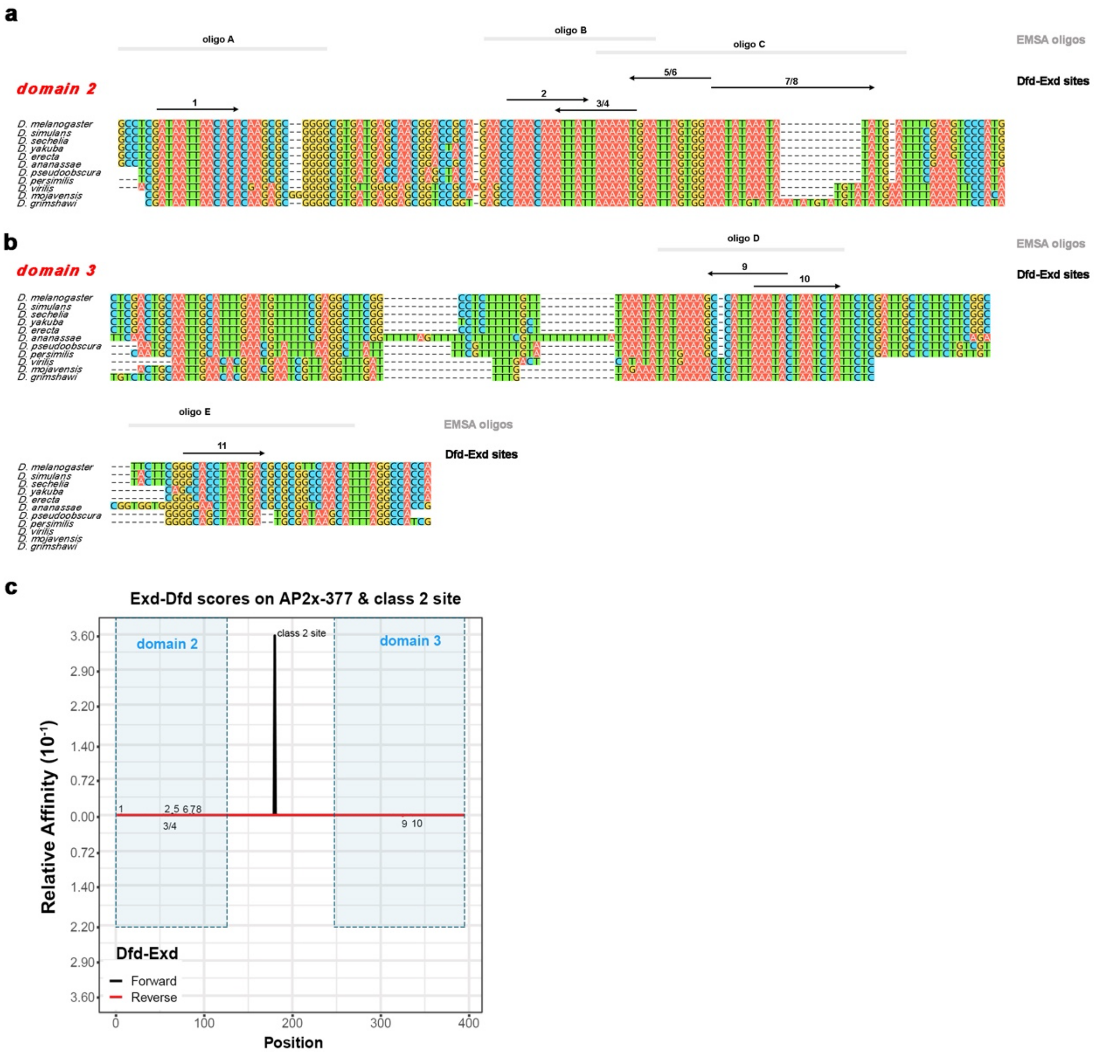

Supplementary Figure 5. **The domains 2 and 3 of the *AP2x-377* enhancer are highly conserved**.

(a, b) Alignment of domains 2 (a) and 3 (b) across *Drosophila* species. The grey bars correspond to the oligonucleotides used for EMSA experiments with black arrows indicating the Dfd-Exd binding sites predicted by the NRLB algorithm[8]. Site 11 (covered by oligo E) was identified by similarity to know characterised low-affinity binding sites. The direction of the arrows indicates the orientation of the Dfd-Exd sites. **(c)** Analysis of the *AP2x-377* enhancer sequence with a Dfd class 2 high-affinity site artificially included in the non-conserved region between domains 2 and 3 using the *No Reads Left Behind* (NRLB) algorithm. This analysis shows that all binding sites present in the *AP2x-377* enhancer are of predicted low affinity in comparison to the Dfd class 2 high-affinity site.

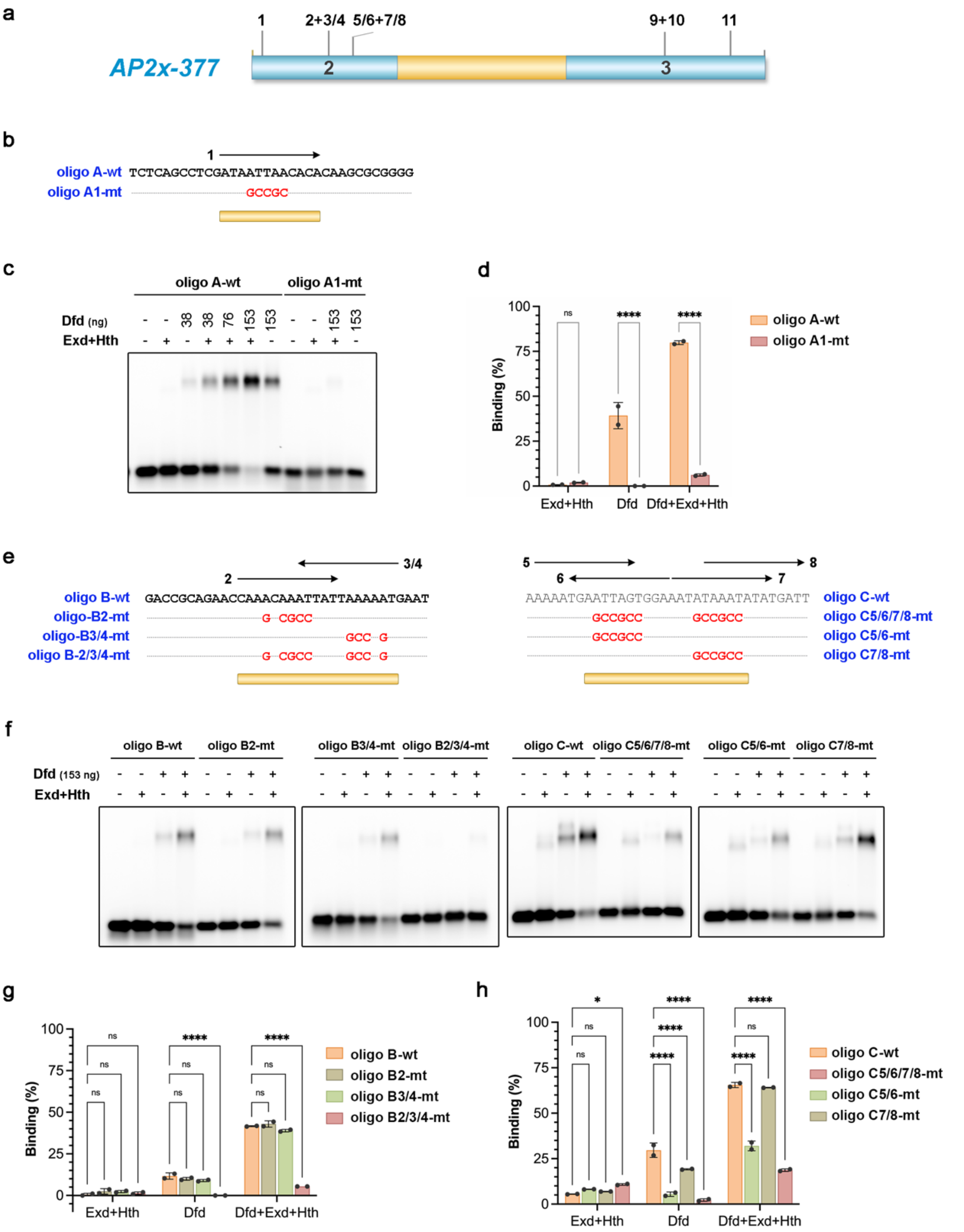

Supplementary Figure 6. Dfd-Exd complexes bind to predicted Dfd-Exd binding sites present in the *AP2x-377* enhancer *in vitro*.

(a) Schematic representation of the *AP2x-377* enhancer with the predicted Dfd-Exd binding sites indicated. **(b, e)** Oligonucleotides (oligo A, oligo B and oligo C) overlapping the *AP2x-377* regions with predicted Dfd-Exd binding sites (site 1 in oligo A (b) and sites 2, 3/4 in oligo B and sites 5, 6, 7, 8 in oligo C (e)). Oligonucleotides were also designed containing mutations to disrupt the predicted sites. **(c, f)** The oligonucleotides shown in (b) and (e) were used to perform EMSAs with Dfd, Exd and

Hth. **(d, g, h)** Quantification of Dfd-Exd relative binding affinities to the predicted Dfd-Exd binding sites present in *AP2x-377* was done by calculating the ratio of bound probe to unbound free probe. The plots indicate the mean and the corresponding standard deviation values. Statistical relevance was tested with the one-way ANOVA test. Yellow bars in (b, e) highlight the Dfd-Exd binding sites in the respective oligos.
ns: non-significant; *: p-value= 0,0122; ****: p-value <0,0001.
Source files are provided in “Source-Data-File_values”.

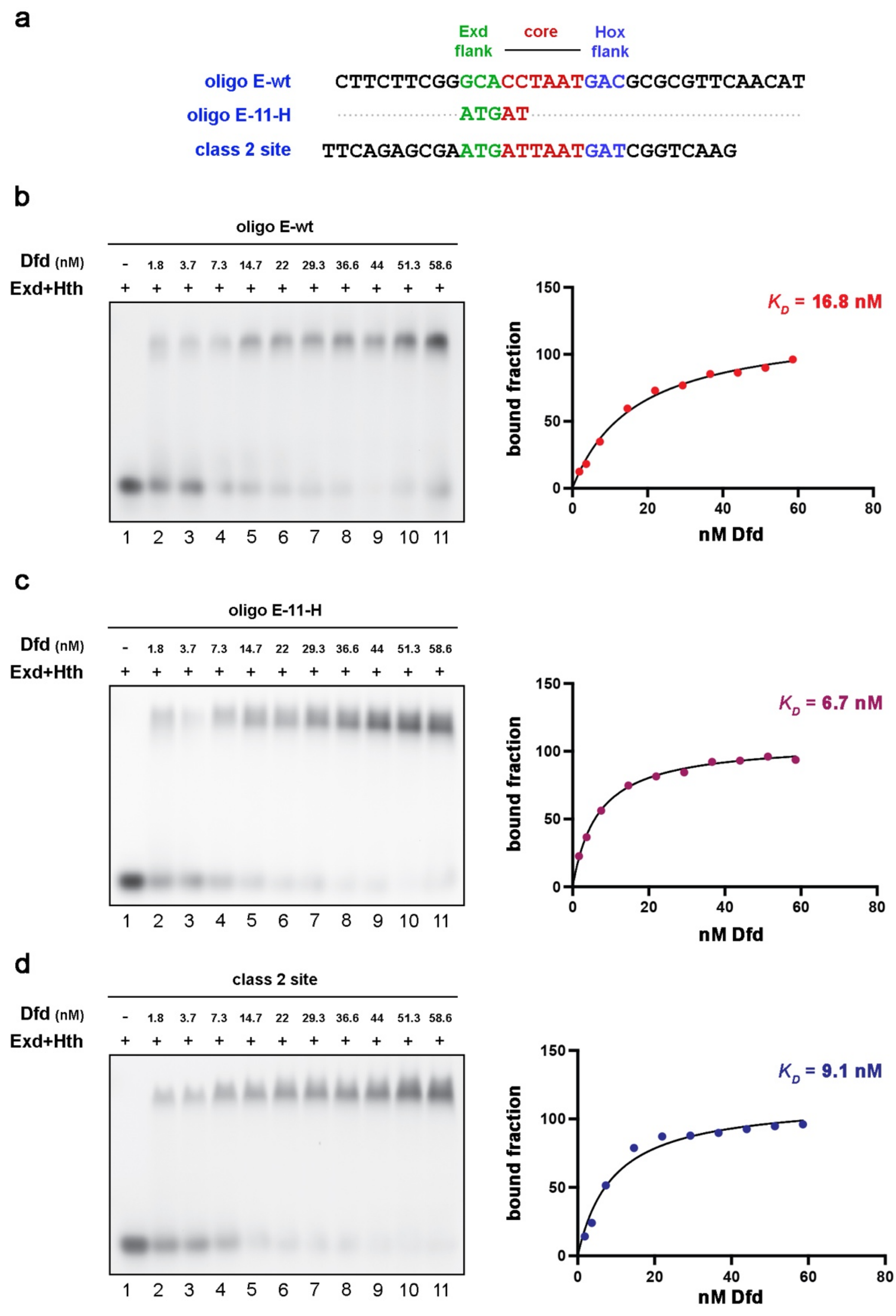

Supplementary Figure 7. Dfd-Exd complexes bind with increased affinity to optimized Dfd-Exd binding sites present in the *AP2x-377* high-affinity enhancers.

(a) Conversion of the *AP2x-377* Dfd-Exd binding site 11 present in oligo E (oligo E-wt) to an optimal class 2 high-affinity Dfd-Exd site (oligo-E-11-H). The lower sequence (class 2 site) shows a high-affinity class 2 Dfd-Exd binding sequence. Nucleotides in

red indicate the Dfd-Exd core sequence, with the Hox and Exd flanking regions in blue and green, respectively. **(b, c, d)** The panels on the left show the EMSAs performed using the oligonucleotides from (a) with different concentrations of Dfd in the presence of constant concentrations of Exd and Hth. The graphs on the right show the quantification of binding affinities of Dfd-Exd complexes to the different oligonucleotides by measuring the respective equilibrium dissociation constants (K_D). Source files are provided in “Source-Data-File_values”.

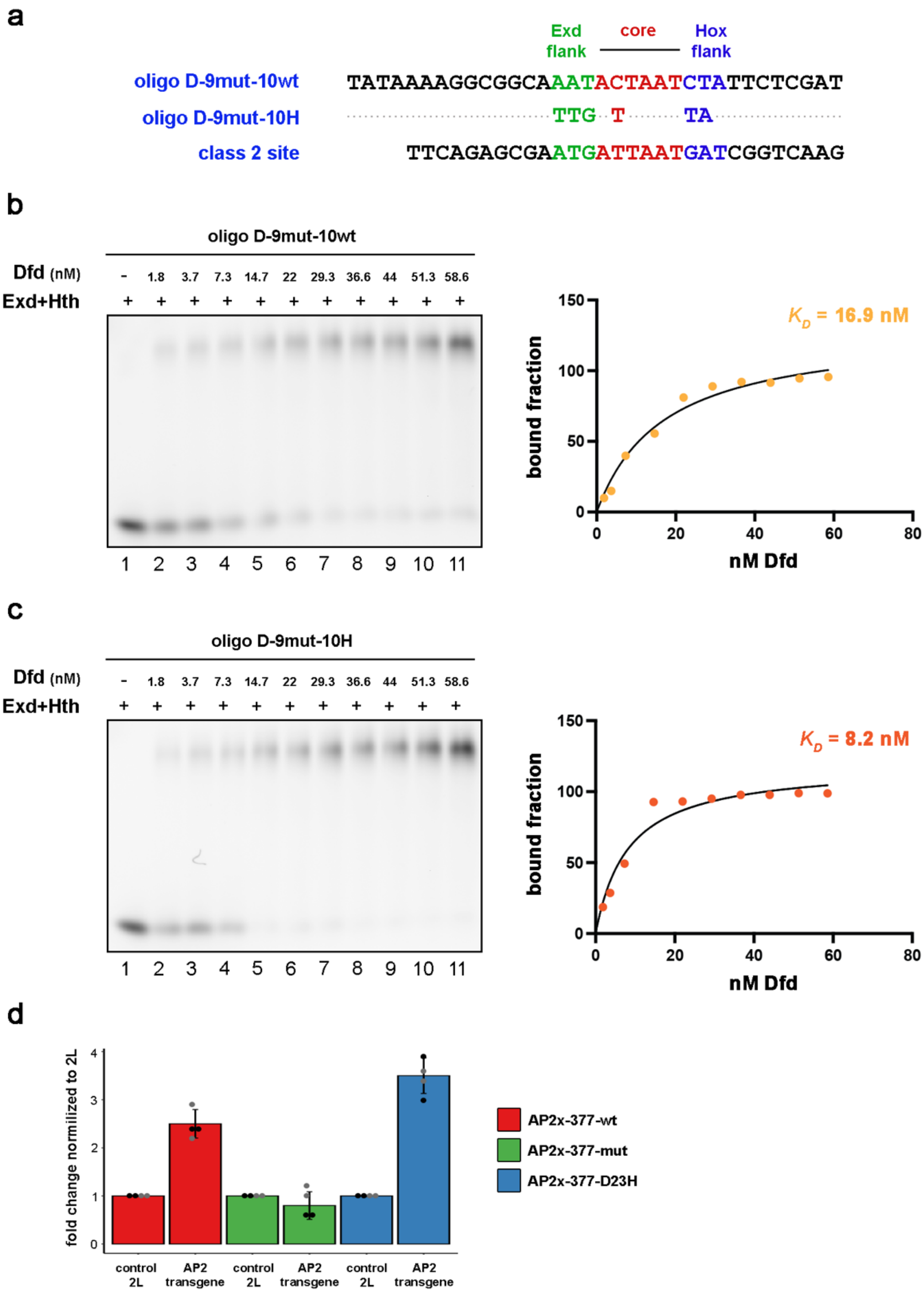

Supplementary Figure 8. **Dfd-Exd complexes bind with increased affinity to optimized Dfd-Exd binding sites present in the *AP2x-377* high-affinity enhancers.**

(a) Conversion of the *AP2x-377* Dfd-Exd binding site 10 of oligo D (oligo D-9mut-10wt) to an optimal class 2 high-affinity Dfd-Exd site (oligo D-9mut-10H). As oligo D contains two Dfd-Exd binding sites, binding site 9 was mutated in order to test only site 10. The lower sequence (class 2 site) shows a high-affinity class 2 Dfd-Exd binding sequence. Nucleotides in red indicate the Dfd-Exd core sequence, with the Hox and Exd flanking

regions in blue and green, respectively. **(b, c)** The panels on the left show the EMSAs performed using the oligonucleotides from (a) with different concentrations of Dfd in the presence of constant concentrations of Exd and Hth. The graphs on the right show the quantification of binding affinities of Dfd-Exd complexes to the different oligonucleotides by measuring the respective equilibrium dissociation constants (K_D). **(d)** Dfd ChIP experiments performed on chromatin extracted from embryos carrying the following transgenes: *AP2x-377-wt*-GAL4, *AP2x-377-mut*-GAL4, and *AP2x-377-D23H*-GAL4. The transgene region and a control region were qPCR amplified using specific primers (see Materials and Methods). The ChIP was performed in two independent replicates, each replicate was tested twice by qPCR. The plots indicate the mean. Dfd binding to the transgene enhancer is 1.4-fold increased in *AP2x-377-D23H*-GAL4 in comparison to *AP2x-377-wt*-GAL4 embryos, while binding is completely abolished when Dfd binding sites are mutated. Black and grey dots indicate different biological replicates.
Source files are provided in “Source-Data-File_values”.

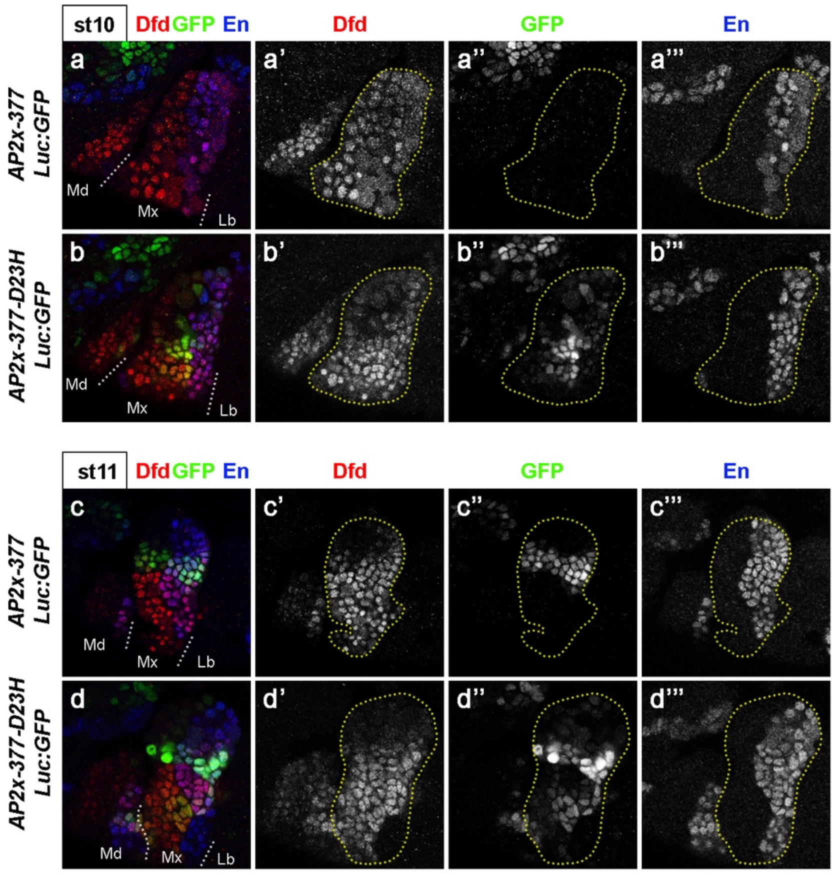

Supplementary Figure 9. **Optimisation of Dfd-Exd binding sites results in spatial and temporal mis-regulation of the *AP2x-377* enhancer**.

(a, b) Activation of *AP2x-377* and *AP2x-377-D23H* enhancers in stage 10 (a) and 11 (b) embryos. Embryos carrying the reporters *AP2x-377-Luc:GFP* (a-a''', c-c''') and *AP2x-377-D23H-Luc:GFP* (b-b''', d-d''') were stained for GFP to determine the activity of both enhancers (green in a, b, c, d, grey in a'', b'', c'', d''). The maxillary segment was labelled by staining the embryos for Dfd (red in a, b, c, d, grey in a', b', c', d') while the posterior maxillary compartment was labelled by En (blue in a, b, c, d, grey in a''', b''', c'', d'''). Md: Mandibular segment; Mx: Maxillary segment; Lb: Labial segment. The yellow dotted lines outline the maxillary segment. White dashed lines indicate the segmental borders.

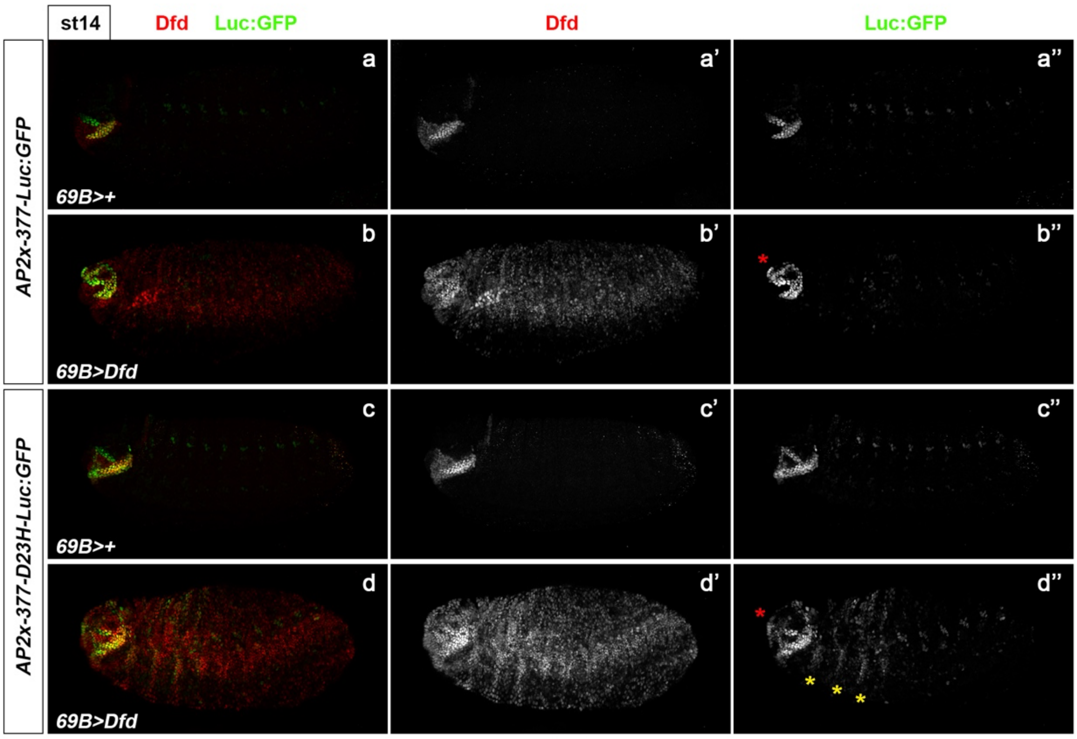

Supplementary Figure 10. Class 2 Dfd-Exd sites are sensitive to Dfd levels. **(a-b’’)** Expression of *AP2x-377-Luc:GFP* in stage 14 embryos in the absence (a-a’’) or presence (b-b’) of ectopically expressed *UAS-Dfd* by means of the *69B-GAL4* ectodermal driver. **(c-d’’)** Expression of *AP2x-377-D23H-Luc:GFP* in stage 14 in the absence (c-c’’) or presence (d-d’’) of ectopically expressed *UAS-Dfd* by means of the *69B-GAL4* ectodermal driver. The activity of *AP2x-377* and *AP2x-377-D23H* was determined by staining embryos for GFP (green in a-d, grey in a’’-d’’). Overexpression of Dfd was determined by staining embryos for Dfd (red in a-d, grey in a’-d’). The red asterisks in (b’’, d’’) indicate ectopic antennal expression, the yellow asterisks in (d’’) highlight ectopic transgene expression in thoracic segments.

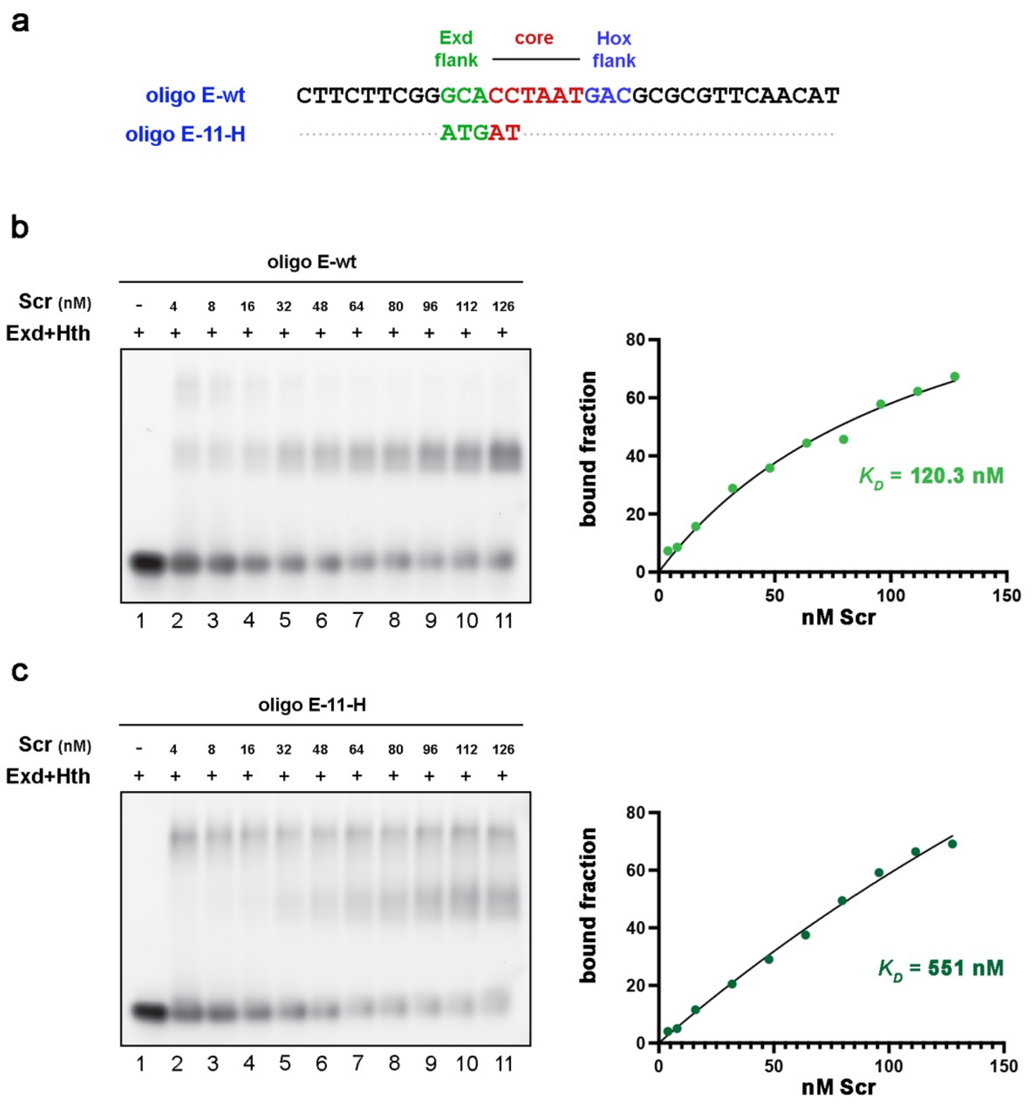

Supplementary Figure 11. **Scr-Exd binds with very low affinity to Dfd-Exd binding sites present in the *AP2x-377* enhancer.**

(a) Representation of the oligonucleotides containing the wild-type *AP2x-377* Dfd-Exd binding site 11 (oligo E-wt) and its optimized high-affinity version (oligo-E-11-H). Nucleotides in red indicate the Dfd-Exd core sequence, with the Hox and Exd flanking regions in blue and green, respectively. **(b, c)** The panels on the left show the EMSAs performed using oligos E-wt and E-11-H with different concentrations of Scr in the presence of constant concentrations of Exd and Hth. The graphs on the right side show the quantification of binding affinities of Scr-Exd complexes to the different oligonucleotides by measuring the respective equilibrium dissociation constants (K_D). Source files are provided in “Source-Data-File_values”.

Supplementary Table 1- Rescue of *AP-2*^{15}- Quantification of *AP-2*^{15} cuticles with ventral cirri

at 25°C			no. Cuticles in mutant with ventral Cirri	Total
♂ *AP2x-377-Gal4/+; AP-2*^{15}*, AbdB*^{M1}*/+*	*x*	♀ *UAS-AP-2/UAS-AP-2, AP-2*^{15}*, AbdB*^{M1}*/TM6B*	10	12
♂ *AP2x-377-D2mt-Gal4/+; AP-2*^{15}*, AbdB*^{M1}*/+*	*x*	♀ *UAS-AP-2/UAS-AP-2, AP-2*^{15}*, AbdB*^{M1}*/TM6B*	12	22
♂ *AP2x-377-D3mt-Gal4/+; AP-2*^{15}*, AbdB*^{M1}*/+*	*x*	♀ *UAS-AP-2/UAS-AP-2, AP-2*^{15}*, AbdB*^{M1}*/TM6B*	5	18
♂ *AP2x-377-D2H-Gal4/+; AP-2*^{15}*, AbdB*^{M1}*/+*	*x*	♀ *UAS-AP-2/UAS-AP-2, AP-2*^{15}*, AbdB*^{M1}*/TM6B*	14	22
♂ *AP2x-377-D3H-Gal4/+; AP-2*^{15}*, AbdB*^{M1}*/+*	*x*	♀ *UAS-AP-2/UAS-AP-2, AP-2*^{15}*, AbdB*^{M1}*/TM6B*	16	28
♂ *AP2x-377-D23H-Gal4/+; AP-2*^{15}*, AbdB*^{M1}*/+*	*x*	♀ *UAS-AP-2/UAS-AP-2, AP-2*^{15}*, AbdB*^{M1}*/TM6B*	10	18

Supplementary Table 2- Rescue of *AP-2*^{15} - Quantification of *AP-2*^{15} cuticles with dorsal cirri

	of cuticles *AP-2*^{15}/ *AP-2*^{15}		
at 25°C	no ventral Cirri	with ventral Cirri	Total
♂ *AP2x-377-Gal4/+; AP-2*^{15}*, AbdB*^{M1}*/+* x ♀ *UAS-AP-2/UAS-AP-2, AP-2*^{15}*, AbdB*^{M1}*/TM6B*	2 / 2	10 / 10	12
♂ *AP2x-377-D2mt-Gal4/+; AP-2*^{15}*, AbdB*^{M1}*/+* x ♀ *UAS-AP-2/UAS-AP-2, AP-2*^{15}*, AbdB*^{M1}*/TM6B*	9 / 9	10 / 10	19
♂ *AP2x-377-D3mt-Gal4/+; AP-2*^{15}*, AbdB*^{M1}*/+* x ♀ *UAS-AP-2/UAS-AP-2, AP-2*^{15}*, AbdB*^{M1}*/TM6B*	12 / 12	5 / 5	17
♂ *AP2x-377-D2H-Gal4/+; AP-2*^{15}*, AbdB*^{M1}*/+* x ♀ *UAS-AP-2/UAS-AP-2, AP-2*^{15}*, AbdB*^{M1}*/TM6B*	8 / 8	14 / 14	22
♂ *AP2x-377-D3H-Gal4/+; AP-2*^{15}*, AbdB*^{M1}*/+* x ♀ *UAS-AP-2/UAS-AP-2, AP-2*^{15}*, AbdB*^{M1}*/TM6B*	12 / 12	7 / 14	26
♂ *AP2x-377-D23H-Gal4/+; AP-2*^{15}*, AbdB*^{M1}*/+* x ♀ *UAS-AP-2/UAS-AP-2, AP-2*^{15}*, AbdB*^{M1}*/TM6B*	8 / 8	9 / 10	18

Supplementary Table 3- PCR reactions used to generate the different AP2x enhancers

	PCR reaction #1		PCR reaction #2		PCR reaction #3		PCR reaction #4	
Insert	Template	primers	Template	primers	Template	primers	Template	primers
AP2x-592	pENTR-AP2x	AP2x-354-Fw + AP2x-496-Rev	-	-	-	-	-	-
AP2x-377	pENTR-AP2x	AP2x-354-Fw + AP2x-731-Rev	-	-	-	-	-	-
AP2x-214	pENTR-AP2x	AP2x-732-Fw + AP2x-496-Rev	-	-	-	-	-	-
AP2x-230	pENTR-AP2x	AP2x-354-Fw + AP2x-584C-Rev	-	-	-	-	-	-
AP2x-268	pENTR-AP2x	AP2x-464-Fw + AP2x-731-Rev	-	-	-	-	-	-
AP2x-152	pENTR-AP2x	AP2x-580-Fw + AP2x-731-Rev	-	-	-	-	-	-

AP2x-109	pENTR-AP2x	AP2x-354-Fw + AP2x-463-Rev	-	-	-	-	-	-
AP2x-377-D2H	pENTR-AP2x	domain-2-Fw + 4.3-wt-Rev	pENTR-AP2x	3.1-High-Fw + 3.2.3.3-high-Rev	PCR #1 +PCR #2	3.1-High-Fw + 4.3-wt-Rev	-	-
AP2x-377-D3H	pENTR-AP2x	AP2x-354-Fw + domain-4-Rev	pENTR-AP2x	4.2-High-Fw + 4.3-High-Rev	PCR #1 +PCR #2	AP2x-354-Fw + 4.3-High-Rev	-	-
AP2x-377-D23H	pENTR-AP2x	3.1-High-Fw + 3.2.3.3-high-Rev	pENTR-AP2x	domain-2-Fw + domain-4-Rev	pENTR-AP2x	4.2-High-Fw + 4.3-High-Rev	PCR #1 +PCR #2 + PCR #3	3.1-High-Fw + 4.3-High-Rev
AP2x-377-D2H-D3mt	pENTR-AP2x	3.1-High-Fw + 3.2.3.3-high-Rev	pENTR-AP2x	domain-2-Fw + domain-4-Rev	pENTR-AP2x	4.1.2-mt-Fw + 4.3-mt-Rev	PCR #1 +PCR #2 + PCR #3	3.1-High-Fw + 4.3-mt-Rev
AP2x-377-D2mt-D3H	pENTR-AP2x	TOPO-B3-mt-Fw + D2-C-Rev	pENTR-AP2x	domain-2-Fw + domain-4-Rev	pENTR-AP2x	4.2-High-Fw + 4.3-High-Rev	PCR #1 +PCR #2 + PCR #3	TOPO-B3-mt-Fw + 4.3-High-Rev
AP2x-377-D2mt	pENTR-AP2x	TOPO-B3-mt-Fw + D2-C-Rev	pENTR-AP2x	domain-2-Fw + 4.3-wt-Rev	PCR #1 +PCR #2	TOPO-B3-mt-Fw + 4.3-wt-Rev	-	-
AP2x-377-D3mt	pENTR-AP2x	AP2x-354-Fw + domain-4-Rev	pENTR-AP2x	4.1.2-mt-Fw + 4.3-mt-Rev	PCR #1 +PCR #2	AP2x-354-Fw + 4.3-mt-Rev	-	-
AP2x-377-D23mt	pENTR-AP2x	TOPO-B3-mt-Fw + D2-C-Rev	pENTR-AP2x	4.1.2-mt-Fw + 4.3-mt-Rev	PCR #1 +PCR #2	domain-2-Fw + domain-4-Rev	PCR #1 +PCR #2 + PCR #3	TOPO-B3-mt-Fw + 4.3-mt-Rev

Supplementary Table 4- List of primers used to generate the different plasmid constructs

Primer	Sequence (5'-3')
AP2x-354-Fw	CACCTCGATAATTAACACACAAGCGCG
AP2x-464-Fw	CACCGAACCGCTGGGGCCACGTGCTTCTAG
AP2x-580-Fw	CACCCAACTCGACTGCAATTGCATTTGA
AP2x-732-Fw	CACCATCGGGCCAGGGGACCAGTGGGTC
domain-2-Fw	AATATAAATATATGATTTTGAAGTCCCATGAACCGC
3.1-High-Fw	CACCTCGTTGATTAATTGTCAAGCGCGGGGCGTGATGAG
4.1.2-mt-Fw	GGCTTCGGCCTCTTTTTGTTTAAATATATAAAAGGCGGCAAATAGCCGCCTATTCTCGATTGCTCTTCTTCGGCTTCTTCGG
4.2-High-Fw	GGCTTCGGCCTCTTTTTGTTTAAATATATAAAAGCCATTATTGATTAATTAATTCTCGATTGCTCTTCTTCGGCTTCTTCGG
TOPO-B3-mt-Fw	CACCTCGATAGCCGCCACACAAGCGCGGGGCGTGATGAG
AP2x-496-Rev	TGAATGCCTAGAAGCACGTGGCCCCAGCG
AP2x-584C-Rev	GTTGGGTGCATGGAGCTGAGAGTTG
AP2x-463-Rev	ATGGGACTTCAAAATCATATATTTATATTTCCA
AP2x-731-Rev	GGTGGCCTAAATGTTGAACGCGCG
domain-4-Rev	CTTTTATATATTTAAACAAAAAGAGGCCGAAGCC
3.2.3.3-high-Rev	GCGGTTCATGGGACTTCAAAATCATATATTTATATTTTGATTAATTAATTTTTAATAATTAATCAAGTTCTGCGGTCCGTTGCTCATCACGCC

D2-C-Rev	GCGGTTCATGGGACTTCAAAATCATATATTTATATTTCCGGCGGCTCATTTTTAATAGGCGGCTTGGTTCTGCGGTCCGTTGCTCATCACGCC
4.3-wt-Rev	GGTGGCCTAAATGTTGAACGCGCGTCATTAGGTGCCCGAAGAAGCCGAAGAAGAGCAATCGAGAATAG
4.3-mt-Rev	GGTGGCCTAAATGTTGAACGCGCGTCGCCGCGTGCCCGAAGAAGCCGAAGAAGAGCAATCGAGAATAG
4.3-High-Rev	GGTGGCCTAAATGTTGAACGCGCGTCATTAATCAACCGAAGAAGCCGAAGAAGAGCAATCGAGAAT
6His-myc-Fw	CACCACCACCACCACCACGAACAAAAGCTTATTTCTGAAGAAGACTTGAATTCACAG
6His-Flag-Fw	CACCACCACCACCACCACGATTACAAGGATGACGACGATAAGGGAATTCGATTGGGGATCCGAATGGC
UAS-KpnI-Rev	TCTAGAGGTACCCTCGAGCCGCGGC

Supplementary Table 5- Primers used to generate double-stranded oligonucleotides used in EMSAs

double-stranded Oligo	single-stranded Oligo (sense)	single-stranded Oligo (anti-sense)
oligo-A-wt	TCTCAGCCTCGATAATTAACACACAAGCGCGGGG	CCCCGCGCTTGTGTGTTAATTATCGAGGCTGAGA
oligo-A-mt	TCTCAGCCTCGATAGCCGCCACACAAGCGCGGGG	CCCCGCGCTTGTGTGGCGGCTATCGAGGCTGAGA
oligo-B-wt	GACCGCAGAACCAAACAAATTATTAAAAATGAAT	ATTCATTTTTAATAATTTGTTTGGTTCTGCGGTC
oligo-B-1-mt	GACCGCAGAACCAAGCCGCCTATTAAAAATGAAT	ATTCATTTTTAATAGGCGGCTTGGTTCTGCGGTC
oligo-B-2-mt	GACCGCAGAACCAAACAAATTATTGCCAGTGAAT	ATTCACTGGCAATAATTTGTTTGGTTCTGCGGTC
oligo-B-12-mt	GACCGCAGAACCAAGCCGCCTATTGCCAGTGAAT	ATTCACTGGCAATAGGCGGCTTGGTTCTGCGGTC
oligo-C-wt	AAAAATGAATTAGTGGAAATATAAATATATGATT	AATCATATATTTATATTTCCACTAATTCATTTTT
oligo-C-1-mt	AAAAATGAGCCGCCGGAAATATAAATATATGATT	AATCATATATTTATATTTCCGGCGGCTCATTTTT
oligo-C-2-mt	AAAAATGAATTAGTGGAAATGCCGCCATATGATT	AATCATATGGCGGCATTTCCACTAATTCATTTTT
oligo-C-12-mt	AAAAATGAGCCGCCGGAAATGCCGCCATATGATT	AATCATATGGCGGCATTTCCGGCGGCTCATTTTT
oligo-C-2-mt-HC	AAAAATGAATTAATGGAAATGCCGCCATATGATT	AATCATATGGCGGCATTTCCATTAATTCATTTTT
oligo-C-2-mt-HCF1	AAAAATGAATTAATCAAAATGCCGCCATATGATT	AATCATATGGCGGCATTTTGATTAATTCATTTTT
oligo-C-2-mt-HCF2	AAAAATTAATTAATGGAAATGCCGCCATATGATT	AATCATATGGCGGCATTTCCATTAATTAATTTTT

oligo-C-2-mt-HCF12	AAAAATTAATTAATCAAAATGCCGCCATATGATT	AATCATATGGCGGCATTTTGATTAATTAATTTTT
oligo-D-wt	TATAAAAGCCATTAAATACTAATCTATTCTCGAT	ATCGAGAATAGATTAGTATTTAATGGCTTTTATA
oligo-D-1-mt	TATAAAAGGCGGCAAATACTAATCTATTCTCGAT	ATCGAGAATAGATTAGTATTTGCCGCCTTTTATA
oligo-D-2-mt	TATAAAAGCCATTAAATAGCCGCCTATTCTCGAT	ATCGAGAATAGGCGGCTATTTAATGGCTTTTATA
oligo-D-12-mt	TATAAAAGGCGGCAAATAGCCGCCTATTCTCGAT	ATCGAGAATAGGCGGCTATTTGCCGCCTTTTATA
oligo-D-1-mt-2-H	TATAAAAGGCGGCATTGATTAATTAATTCTCGAT	ATCGAGAATTAATTAATCAATGCCGCCTTTTATA
oligo-E-wt	CTTCTTCGGGCACCTAATGACGCGCGTTCAACAT	ATGTTGAACGCGCGTCATTAGGTGCCCGAAGAAG
oligo-E-mt	CTTCTTCGGGCACGCGGCGACGCGCGTTCAACAT	ATGTTGAACGCGCGTCGCCGCGTGCCCGAAGAAG
oligo-E-11-H	CTTCTTCGGATGATTAATGACGCGCGTTCAACAT	ATGTTGAACGCGCGTCATTAATCATCCGAAGAAG
oligo-class-2-site	TTCAGAGCGAATGATTAATGATCGGTCAAG	CTTGACCGATCATTAATCATTCGCTCTGAA
