# Supplementary material for: Specificity of the Hox member Deformed is determined by transcription factor levels and binding site affinities
Source: Nat Commun. 2022 Aug 26;13:5037. doi: 10.1038/s41467-022-32408-8 (PMC9418327; doi:10.1038/s41467-022-32408-8)
Supplement: Supplementary file 3 — Reporting Summary [file 41467_2022_32408_MOESM3_ESM.pdf]

nature portfolio

Corresponding author(s): Ingrid Lohmann

Last updated by author(s): 13/07/22

Reporting Summary

Nature Portfolio wishes to improve the reproducibility of the work that we publish. This form provides structure for consistency and transparency in reporting. For further information on Nature Portfolio policies, see our Editorial Policies and the Editorial Policy Checklist.

Statistics

For all statistical analyses, confirm that the following items are present in the figure legend, table legend, main text, or Methods section.

n/a	Confirmed	
☒	☐	The exact sample size (n) for each experimental group/condition, given as a discrete number and unit of measurement
☐	☒	A statement on whether measurements were taken from distinct samples or whether the same sample was measured repeatedly
☐	☒	The statistical test(s) used AND whether they are one- or two-sided<br>*Only common tests should be described solely by name; describe more complex techniques in the Methods section.*
☒	☐	A description of all covariates tested
☒	☐	A description of any assumptions or corrections, such as tests of normality and adjustment for multiple comparisons
☒	☐	A full description of the statistical parameters including central tendency (e.g. means) or other basic estimates (e.g. regression coefficient) AND variation (e.g. standard deviation) or associated estimates of uncertainty (e.g. confidence intervals)
☒	☐	For null hypothesis testing, the test statistic (e.g. F, t, r) with confidence intervals, effect sizes, degrees of freedom and P value noted<br>*Give P values as exact values whenever suitable.*
☒	☐	For Bayesian analysis, information on the choice of priors and Markov chain Monte Carlo settings
☒	☐	For hierarchical and complex designs, identification of the appropriate level for tests and full reporting of outcomes
☒	☐	Estimates of effect sizes (e.g. Cohen's d, Pearson's r), indicating how they were calculated

Our web collection on statistics for biologists contains articles on many of the points above.

Software and code

Policy information about availability of computer code

Data collection	No genome-wide data was produced in the study and no other large scale date collection was performed.
Data analysis	No Reads Left Behind (NRLB) algorithm (https://github.com/BussemakerLab/NRLB) version 1, published in 2018

For manuscripts utilizing custom algorithms or software that are central to the research but not yet described in published literature, software must be made available to editors and reviewers. We strongly encourage code deposition in a community repository (e.g. GitHub). See the Nature Portfolio guidelines for submitting code & software for further information.

Data

Policy information about availability of data

All manuscripts must include a data availability statement. This statement should provide the following information, where applicable:

- Accession codes, unique identifiers, or web links for publicly available datasets
- A description of any restrictions on data availability
- For clinical datasets or third party data, please ensure that the statement adheres to our policy

The data availability statement is listed in the manuscript and the raw data generated in the study is available in the two data source files (data_source_value, data_source_gel). In this study was no genome-wide data produced or used.

Field-specific reporting

Please select the one below that is the best fit for your research. If you are not sure, read the appropriate sections before making your selection.

☒ Life sciences ☐ Behavioural & social sciences ☐ Ecological, evolutionary & environmental sciences

For a reference copy of the document with all sections, see nature.com/documents/nr-reporting-summary-flat.pdf

Life sciences study design

All studies must disclose on these points even when the disclosure is negative.

Sample size	A suitable sample size was used for intensity measurements of 8-10 individuals embryos. There was no sample size calculation performed and in each experiment the sample size is annotated in the figure legend.
Data exclusions	no data was excluded
Replication	Two replicates were used for the ChIP experiment as annotated in the Material&Methods as well as in the figure legend. Both all attempts at replications were successful and showed similar results.
Randomization	Randomization was not relevant for the study
Blinding	Blinding was not relevant for the study

Reporting for specific materials, systems and methods

We require information from authors about some types of materials, experimental systems and methods used in many studies. Here, indicate whether each material, system or method listed is relevant to your study. If you are not sure if a list item applies to your research, read the appropriate section before selecting a response.

Materials & experimental systems

n/a	Involved in the study	
☐	☒	Antibodies
☒	☐	Eukaryotic cell lines
☒	☐	Palaeontology and archaeology
☐	☒	Animals and other organisms
☒	☐	Human research participants
☒	☐	Clinical data
☒	☐	Dual use research of concern

Methods

n/a	Involved in the study	
☒	☐	ChIP-seq
☒	☐	Flow cytometry
☒	☐	MRI-based neuroimaging

Antibodies

Antibodies used	The following primary antibodies were used: guinea pig anti-Dfd (1:500) (Velten et al., 2022), mouse anti-En 4D9 (1:2.5) (Developmental Studies Hybridoma Bank), rabbit anti-Paired (1:1000, from Markus Noll), rabbit anti-Dll (1:100, from Sean Carroll), mouse anti-βGal (1:1000, Ref: Z3781, Lot: 0000393241, Promega), rabbit anti-GFP (1:300, Ref: A11122, Lot: 2083201, Invitrogen) and rat anti-RFP (5F8) (1:100, ref: 5f8-100, Lot: 90228062AB-15, Chromotek).<br>rabbit anti-Paired and rabbit anti-Dll have no further reference or lot numbers, since they were individually made and distributed by the annotated Labs or people.<br>Secondary antibodies were used according to the animal of the first and were conjugates with different fluorophores, Alexa Fluor 488, Cy3 and Alexa Fluor 647 and obtained from Jackson ImmunoResearch Laboratories (1:200).
Validation	no validation of the antibodies was performed, they were used as recommended by the supplier

Animals and other organisms

Policy information about studies involving animals; ARRIVE guidelines recommended for reporting animal research

Laboratory animals	Laboratory organism: Drosophila melanogaster<br>We used different Drosophila fly lines for genetic experiments all are listed in the Material&Methods as well as in Table 1b and c. The study did not require any determination about sex and age.
Wild animals	Not used
Field-collected samples	No field collection was performed in this study

Ethics oversight | No Ethical approval were needed for the study

Note that full information on the approval of the study protocol must also be provided in the manuscript.
